# Size-Based Routing Policies: Non-Asymptotic Analysis and Design of Decentralized Systems †

**DOI:** 10.3390/s21082701

**Published:** 2021-04-12

**Authors:** Eitan Bachmat, Josu Doncel

**Affiliations:** 1 Department of Computer Science, Ben-Gurion University, Beer-Sheva 84105, Israel; ebachmat@bgu.ac.il; 2 Mathematics Department, University of the Basque Country, UPV/EHU, 48940 Leioa, Spain

**Keywords:** parallel servers, size-based routing, queuing theory

## Abstract

Size-based routing policies are known to perform well when the variance of the distribution of the job size is very high. We consider two size-based policies in this paper: Task Assignment with Guessing Size (TAGS) and Size Interval Task Assignment (SITA). The latter assumes that the size of jobs is known, whereas the former does not. Recently, it has been shown by our previous work that when the ratio of the largest to shortest job tends to infinity and the system load is fixed and low, the average waiting time of SITA is, at most, two times less than that of TAGS. In this article, we first analyze the ratio between the mean waiting time of TAGS and the mean waiting time of SITA in a non-asymptotic regime, and we show that for two servers, and when the job size distribution is Bounded Pareto with parameter α=1, this ratio is unbounded from above. We then consider a system with an arbitrary number of servers and we compare the mean waiting time of TAGS with that of Size Interval Task Assignment with Equal load (SITA-E), which is a SITA policy where the load of all the servers are equal. We show that in the light traffic regime, the performance ratio under consideration is unbounded from above when (i) the job size distribution is Bounded Pareto with parameter α=1 and an arbitrary number of servers as well as (ii) for Bounded Pareto distributed job sizes with α∈(0,2)\{1} and the number of servers tends to infinity. Finally, we use the result of our previous work to show how to design decentralized systems with quality of service constraints.

## 1. Introduction

We explore the performance of a system composed of parallel servers in which the job size distribution satisfies the heavy-tailed property. This property means that the arrival of extremely large jobs occurs with a non-negligible probability and, therefore, a very small portion of the jobs account for half of the system load. For instance, jobs submitted to modern data centers follow a heavy-tailed distribution [1]. Consequently, if we model the servers of data centers as First-Come-First-Served (FCFS) queues, the presence of very long jobs might cause a substantial performance degradation as many short jobs must wait behind a large job for a long time. This problem is solved by using size-based routing policies, in which job sizes are divided into intervals. We consider that two popular size-based routing policies, the Size Interval Task Assignment (SITA), which designates an interval to each server and jobs whose size are in the interval, are executed on the corresponding server. The second size-based routing policy is called Task Assignment with Guessing Size (TAGS). Under this policy, all incoming jobs are routed to server 1 and, for each server *i*, if its execution time in server *i* time exceeds a given server-assigned threshold $s_{i}$, it is stopped and placed in the last position of the queue of server i+1. As SITA assumes that the size of tasks is known and routes directly to the end server, while TAGS incurs overheads, it is clear that SITA outperforms TAGS. An important result comparing the performance of these two policies [2] states that in the asymptotic regime, where the ratio of the largest to shortest job tends to infinity and the system load is fixed and low, the average waiting time of SITA with optimal intervals is, at most, two times that of TAGS with optimal intervals.

The main contributions of this article are summarized as follows.

- We provide a non-asymptotic comparison of the mean waiting time of SITA and TAGS. We analyze a system with two servers and a job size distribution that follows the Bounded Pareto distribution with the tail parameter one. We provide a job size distribution-dependent lower bound on the ratio of average waiting times between the TAGS and SITA systems at very low rates. We compute a good estimate for the bound when the job size is an arbitrary Bounded Pareto distribution and show that unlike the case of a fixed and low load, when the load is low but not fixed, the performance ratio is unbounded. We also analyze numerically this performance ratio for bounded Pareto distributions with different tail parameters.
- For any number of servers K≥2, we compare the performance of the TAGS system with the Size Interval Task Assignment with Equal load (SITA-E) system, i.e., with a SITA policy in which the thresholds are set to load balance the servers. We analyze this system in light traffic, and we show that in this regime, asymptotically, the average waiting times of TAGS with optimal intervals and of a single server coincide. Consequently, we can use the results of [3] in which they compare the performance of SITA-E and a single server and conclude that the ratio of the average waiting time of the TAGS and SITA-E system is unbounded in a wide range of cases: (i) when the job size distribution Bounded Pareto with α=1 and K≥2, and (ii) when K→∞ and the job size distribution is Bounded Pareto with α∈(0,2)\{1}.

The stability condition for TAGS systems [2] says that the arrival rate times and the mean job size must be less than a critical load that depends on the job size distribution. Let us note that this stability condition does not depend on the number of servers present in the system. Therefore, if we increase the number of servers and the arrival rate proportionally in a TAGS system, the system will become unstable, which is not the case for other routing policies such as Bernoulli or SITA [4]. Therefore, for the design of a system with a large number of servers it is preferable to consider a decentralized system, as in [3], in which the system is divided into *n* load balanced and equal sized groups which can operate under either SITA or TAGS. The goal is to elucidate the maximum number of groups that can operate under TAGS so as to satisfy a quality of service constraint that states that the average waiting time of the system does not exceed a given threshold. As the performance of TAGS and SITA is very difficult to analyze in a precise manner, we focus on a suboptimal, yet reasonable solution which uses the results of [2].

A conference version of this paper appeared in [5].

The rest of the article is organized as follows. In Section 2, we present the related work. In Section 3, we describe the model we study. In Section 4, we perform the non-asymptotic comparison of the performance of TAGS and SITA for two queues, and in Section 5 we consider asymptotically an arbitrary number of queues. In Section 6, we explore how to design a decentralized system with our quality of service constraints. We discuss the results and conclude in Section 7.

## 2. Related Work

Modern communication systems integrate distributed computing architectures, in which jobs are processed in parallel. Therefore, many researchers have studied how to optimally balance the load in a system with parallel servers, see in [6]. Most of the analysis assumes that the state of the servers is always known. A large class of policies which utilizes this assumption is known as the SQ(d) framework. Policies in this family operate as follows: for each incoming job, d≥2 servers are picked uniformly at random and their states are observed. The job is routed to the server whose state (number of packets or the workload, for instance) is minimal. This family of routing policies has been studied extensively as such policies often perform well [7,8,9,10,11]. However, it is important to note that in [12], it was shown that when the variance of the job size distribution is very large, SQ(d) policies are not optimal. The size-based routing policies which we consider often have good performance under precisely such circumstances.

Size-based routing splits the service time distribution into intervals. By using these intervals, when the servers are FCFS, short jobs do not wait for a long time behind a long job. This is a clear advantage with respect to other routing policies that have been studied in the literature such as Bernoulli routing, for instance.

The first size-based routing policy presented in the literature was the SITA policy [13]. Under this policy, each host receives jobs whose job size is in a designated range [13]. This implies a reduction in the variability of the jobs executed in the servers. The authors of [14] provide an interesting result that shows that there exists a SITA policy (choice of intervals) that minimizes mean response time when the state of the servers cannot be observed; the servers scheduling discipline is FCFS and the size of jobs is available. The analytical expression for the optimal thresholds of a SITA policy is not known in general, even for a system with two servers. Therefore, some authors have studied alternatives such as the SITA-E policy, where the intervals are chosen to equalize the load in all the servers [3,15]. Asymptotic analysis of the optimal thresholds of a SITA policy for Bounded Pareto distributions has been carried out in [16,17] and for a large number of servers in [4]. The performance of SITA policies with two servers has been studied in [18], where the authors provide conditions under which the load should be unbalanced to minimize system performance. In [19], the authors compare the SITA policy with that of Least-Work-Left when the coefficient of variation of job sizes is large and show that the Least-Work-Left policy outperforms SITA in several scenarios.

The TAGS policy was introduced in [20] as a size-based routing policy in the setting where the sizes of incoming jobs does not need to be known. The authors of [2,21] prove that for Bounded Pareto distributions, when the ratio between the largest job size and the shortest job size tends to infinity and the system load is fixed and less than one, the ratio between the average waiting times of TAGS and SITA systems with optimal intervals is at most two. This result means that, in that regime, the penalty for not knowing the job size of incoming tasks is, at most, 2. In this work, we address a similar question but in a non-asymptotic regime.

## 3. Model Description

### 3.1. Notations

We investigate a parallel server system with *K* homogeneous servers. The servers are modeled as FCFS queues. Jobs arrive to the system following a Poisson distribution with rate λ. The job size is assumed to be given by a sequence of i.i.d. random variables, whose distribution we denote by *X*. We denote by E[X] the average job size and by F(s)=P[X<s] the cumulative distribution function of the job size distribution. We assume that F(·) is differentiable, and we let $f\left(s\right)=\frac{dF(s)}{ds}$ be the density function of the job size distribution. We assume that the size of the smallest job is equal to one and of the largest job is *r*, where r>1. The system load is denoted by ρ=λE[X].

### 3.2. TAGS Routing

We consider the TAGS routing policy. We assume that the servers of the system are labeled from 1 to *K*. Let $s_{0}=1$ and $s_{K}=r$. The set of parameters of the policy (intervals) is given by a vector of K−1 cut-off values $s=(s_{1},s_{2},\ldots ,s_{K-1})$ verifying that $x_{S}=s_{0}<s_{1}<s_{2}<\ldots <s_{K-1}<s_{K}=x_{L}$.

All jobs are sent to server 1. If the job completes before $s_{1}$ time units, the job leaves the system; otherwise, the job is stopped after a runtime of $s_{1}$ and is sent to the end of the buffer of server 2, where execution starts from scratch and the process repeats. Thus, for server *i*, jobs that are executed in that server have already been executed in servers 1,2,…,i−2 and i−1 for $s_{1},\ldots ,s_{i-1}$ time units, respectively. If a job at server *i* completes before $s_{i}$ time units, it leaves the system, and if not, it is terminated after $s_{i}$ units of time and placed at the last position of the buffer of the next server. Finally, at the last server, *K*, jobs always run to completion.

We denote by $W^{TAGS}\left(s\right)$ the waiting time of jobs for TAGS routing with vector of cut-offs s. In this work, we denote by $s_{T}$ the optimal vector of cut-offs,

$$s_{T}=\underset{s}{arg min}E\left[W^{TAGS}\left(s\right)\right].$$

### 3.3. SITA Routing

We consider the SITA routing policy. Let $s_{0}=1$ and $s_{K}=r$. Under the SITA policy, the servers are also labeled from 1 to *K* and, as in the TAGS policy, the set of parameters is a vector of thresholds $s=(s_{1},s_{2},\ldots ,s_{K-1})$ satisfying $x_{S}=s_{0}<s_{1}<s_{2}<\ldots <s_{K-1}<s_{K}=x_{L}$. However, the SITA policy uses the job size of incoming tasks to perform the routing. Therefore, jobs ranging in size between $s_{i-1}$ and $s_{i}$ are sent directly to the *i*th server where they complete service.

We denote by $W^{SITA}\left(s\right)$ the waiting time of tasks when the system operates the SITA routing with the vector of thresholds s. The optimal vector of the cut-offs is denoted by $s^{*}$, i.e.,

$$s^{*}=\underset{s}{arg min}E\left[W^{SITA}\left(s\right)\right].$$

We will sometimes be interested in a SITA policy whose thresholds $s_{1},\ldots ,s_{K-1}$ satisfy that the load of all the servers is the same, i.e.,

$$\int_{s_{i-1}}^{s_{i}}xf\left(x\right)dx=\int_{s_{i}}^{s_{i+1}}xf\left(x\right)dx,$$

for all i=2,…,K−1. This policy is called SITA-E, and in this work, we denote by $E[W^{SITA-E}]$ the mean waiting time of incoming jobs under this policy.

**Remark** **1** (Advantages and Disadvantages of TAGS and SITA)**.** *Before going further, it is worth explaining the major advantages and disadvantages of the presented routing policies. First, we would like to remark that both are open loop policies, i.e., they do not require information exchange between the servers and the dispatcher. This is an important advantage with respect to other routing policies such as Join the Shortest Queue, for instance. Moreover, SITA and TAGS are size-based routing policies, i.e., they both use cut-offs to determine how jobs are executed in the servers. The main advantage of SITA is the optimality result in [14]. However, SITA routing uses the size of incoming tasks to assign jobs to servers. Finally, we remark that TAGS do not use the information of the size of incoming jobs to balance the load.*

### 3.4. Bounded Pareto Distributions

Let r>1 and $a=\frac{1}{r}$. If 1≤s≤r, then a distribution follows the Bounded Pareto distribution with parameters 1, *r*, and α if its density function is

$$f\left(s\right)=\frac{\alpha s^{-\alpha -1}}{\left(1-a^{\alpha }\right)},$$

and f(s)=0 otherwise. Note that this distribution consists of the Pareto distribution with tail parameter α, but restricted to a bounded domain, i.e., 1≤s≤r. The cumulative distribution function is given by

$$F\left(s\right)=\left\{\begin{array}{cc} 0, & s\leq 1,\\ \frac{1-{\left(1/s\right)}^{\alpha }}{1-a^{\alpha }}, & 1\leq s\leq r,\\ 1, & s\geq r. \end{array}\right.$$

When α≠1, it is easy to see that

(1) $$E\left[X\right]=\frac{\alpha }{\alpha -1}\frac{1-a^{\alpha -1}}{1-a^{\alpha }}$$

whereas when α=1

(2) $$E\left[X\right]=\frac{\ln(r)}{1-\frac{1}{r}}.$$

We remark that the distributions under consideration when r is large and 0<α<2 are known to model well job size distributions with large variability [22]. Another interesting property of these distributions is that when α=−1, they coincide with the uniform distribution on the interval [1,r].

### 3.5. Application of This Theory to the Result

In this article, we are interesting in investigating the following ratio:

$$\frac{E[W^{TAGS}\left(s_{T}\right)]}{E[W^{SITA}\left(s^{*}\right)]}.$$

The analysis of this ratio can be seen as the penalty for not knowing the size of incoming tasks. The authors of [2] showed that, when *r* is large, this ratio is upper-bounded by 2. Therefore, they conclude that the penalty for not knowing the size of incoming jobs is, at most, 2 in the regime they consider. In this work, we also study this ratio but in a different regime. In Section 4, we consider a system with two queues, whereas in Section 5 an arbitrary number of queues.

## 4. Performance Comparison of TAGS and Optimal SITA with K=2 Servers

We study the ratio of the mean waiting times of TAGS and SITA for Bounded Pareto distributions when *r* is finite. We recall that Theorem 9 in [2] says that the aforementioned performance ratio is upper-bounded by 2 when the system is at a fixed low load and *r* tends to infinity. In this section, we consider a system with two servers, and we show that this ratio is unbounded from above. We first provide an analysis for the case of Bounded Pareto with α=1. Then, we present numerical experiments that show that the ratio is also unbounded from above for Bounded Pareto-distributed jobs sizes with α≠1.

### 4.1. Performance of the Optimal TAGS System

**Figure 1:**
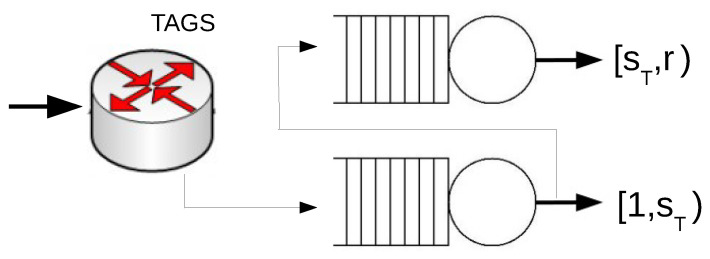
A system with two servers that operates under Task Assignment with Guessing Size (TAGS).

We consider a system with two servers operating under the TAGS policy (see Figure 1). In a system with two servers, the vector of thresholds s is reduced to a unique value; therefore, we denote by $E[W^{TAGS}\left(s\right)]$ the mean waiting time of TAGS for the threshold *s*. Here, we denote by $s_{T}$ the threshold value that minimizes the mean waiting time of the TAGS system, i.e.,

$$s_{T}=\underset{s}{arg min}E\left[W^{TAGS}\left(s\right)\right].$$

From the definition of the TAGS system, it follows that

(3) $$\begin{array}{cc} E[W^{TAGS}\left(s_{T}\right)]= & E\left[W_{1}^{TAGS}\left(s_{T}\right)\right]+\left(\int_{s_{T}}^{r}f\left(x\right)dx\right)s_{T}+\left(\int_{s_{T}}^{r}f\left(x\right)dx\right)E\left[W_{2}^{TAGS}\left(s_{T}\right)\right], \end{array}$$

where $E[W_{i}^{TAGS}\left(s_{T}\right)]$ is the mean waiting time of jobs at server *i* when the threshold value is $s_{T}$.

### 4.2. Performance of the Optimal SITA System

**Figure 2:**
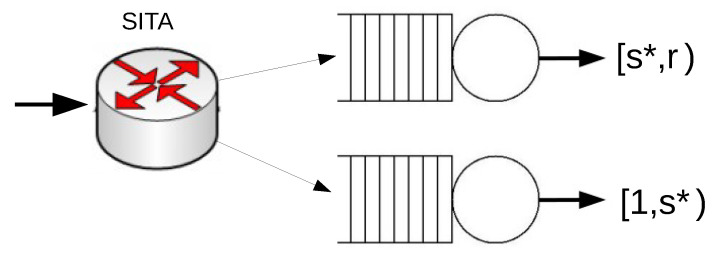
A system with two servers that operates under Size Interval Task Assignment (SITA).

We now consider a system with two servers operating under the SITA policy (see Figure 2). For SITA routing, in a system with two servers, the vector of thresholds s is also reduced to a unique value. In this case, we denote by $E[W^{SITA}\left(s\right)]$ the mean waiting time of the SITA routing system with the threshold *s*. We let $s^{*}$ be the value of *s* such that the mean waiting time of the SITA system is minimized, i.e.,

$$s^{*}=\underset{s}{arg min}E\left[W^{SITA}\left(s\right)\right].$$

The mean waiting time of jobs under the SITA policy with cutoff $s^{*}$ is given by

(4) $$\begin{array}{c} E\left[W^{SITA}\left(s^{*}\right)\right]=\left(\int_{1}^{s^{*}}f\left(x\right)dx\right)E\left[W_{1}^{SITA}\left(s^{*}\right)\right]+\left(\int_{s^{*}}^{r}f\left(x\right)dx\right)E\left[W_{2}^{SITA}\left(s^{*}\right)\right], \end{array}$$

where $E[W_{i}^{SITA}\left(s^{*}\right)]$ is the mean waiting time of jobs at server *i*.

### 4.3. Bounded Pareto Distribution with α=1

We consider the Bounded Pareto distribution with α=1 and compare the mean waiting time of a system with two servers operating under TAGS routing with that of a system with two servers operating under SITA routing. In the following result, we provide a lower-bound for the average waiting time of the TAGS system.

**Lemma** **1.** *For the Bounded Pareto distribution with α=1, if λr<1,*

$$E[W^{TAGS}\left(s_{T}\right)]>\lambda r.$$

**Proof.**  It follows from (3) that

$$\begin{array}{cc} E[W^{TAGS}\left(s_{T}\right)]\geq & E\left[W_{1}^{TAGS}\left(s_{T}\right)\right]+\left(\int_{s_{T}}^{r}f\left(x\right)dx\right)s_{T}. \end{array}$$

For the Bounded Pareto distribution with α=1, we have that

$$\begin{array}{cc} E\left[W_{1}^{TAGS}\left(s_{T}\right)\right]+\left(\int_{s_{T}}^{r}f\left(x\right)dx\right)s_{T} & =\frac{\lambda (s_{T}-1)}{2\left(1-\rho \right)\left(1-\frac{1}{r}\right)}+\frac{1-\frac{s_{T}}{r}}{1-\frac{1}{r}}\\ & \geq \frac{\lambda (s_{T}-1)}{2(1-\frac{1}{r})}+\frac{1-\frac{s_{T}}{r}}{1-\frac{1}{r}}\\ & =\frac{s_{T}\left(\lambda -\frac{1}{r}\right)+2-2\lambda }{2(1-\frac{1}{r})}. \end{array}$$

If λr<1, then $\frac{x(\lambda -\frac{1}{r})+2-2\lambda }{2(1-\frac{1}{r})}$ decreases with *x*. Thus,

$$\begin{array}{cc} \frac{s_{T}\left(\lambda -\frac{1}{r}\right)+2-2\lambda }{2(1-\frac{1}{r})} & \geq \frac{r(\lambda -\frac{1}{r})+2-2\lambda }{2(1-\frac{1}{r})}=\frac{\lambda (r-2)+1}{2(1-\frac{1}{r})}>\frac{\lambda (r-2)+\lambda r}{2(1-\frac{1}{r})}=\lambda r. \end{array}$$

□

Note that, unlike previous work [2], we do not need to assume Poisson arrivals to all the servers in the above result. In the following result, we estimate, non-asymptotically, the mean waiting time of the SITA system.

**Lemma** **2.** *For the Bounded Pareto distribution with α=1, when λr<1 and r is large,*

$$E\left[W^{SITA}\left(s^{*}\right)\right]\leq \frac{\lambda {\left(\sqrt{r}-1\right)}^{2}}{\sqrt{r}{\left(1-\frac{1}{r}\right)}^{2}}.$$

**Proof.**  We first note that the load of each server is upper bounded by ρ. Therefore, for the Bounded Pareto distribution with α=1, we have that

$$E\left[W_{1}^{SITA}\left(s^{*}\right)\right]\leq \frac{\lambda (s^{*}-1)}{2(1-\rho )(1-(1/r))}$$

and

$$E\left[W_{2}^{SITA}\left(s^{*}\right)\right]\leq \frac{\lambda (r-s^{*})}{2(1-\rho )(1-(1/r))},$$

where ρ=λln(r). We now note that

$$\lambda r<1\Leftrightarrow \lambda \ln\left(r\right)<\frac{\ln(r)}{r}.$$

When *r* is large, $\frac{\ln(r)}{r}$ tends to zero and ρ=λln(r); it follows that ρ tends to zero when λr<1. As a result,

$$E\left[W_{1}^{SITA}\left(s^{*}\right)\right]\leq \frac{\lambda (s^{*}-1)}{2(1-(1/r))}$$

and

$$E\left[W_{2}^{SITA}\left(s^{*}\right)\right]\leq \frac{\lambda (r-s^{*})}{2(1-(1/r))}.$$

We know from [18] that for the Bounded Pareto distribution with α=1, $s^{*}$ balances the load of both servers, and therefore

$$\int_{1}^{s^{*}}f\left(x\right)dx=\int_{s^{*}}^{r}f\left(x\right)dx\Leftrightarrow s^{*}=\sqrt{r}.$$

As a result,

$$E\left[W_{1}^{SITA}\left(s^{*}\right)\right]\leq \frac{\lambda (\sqrt{r}-1)}{2(1-(1/r))}$$

and

$$E\left[W_{2}^{SITA}\left(s^{*}\right)\right]\leq \frac{\lambda (r-\sqrt{r})}{2(1-(1/r))}.$$

Therefore, from (4), it follows that

$$\begin{array}{cc} E[W^{SITA}\left(s^{*}\right)] & \leq \left(\int_{1}^{s^{*}}f\left(x\right)dx\right)\frac{\lambda (\sqrt{r}-1)}{2(1-(1/r))}+\left(\int_{s^{*}}^{r}f\left(x\right)dx\right)\frac{\lambda (r-\sqrt{r})}{2(1-(1/r))}\\ \; & =\left(\int_{1}^{\sqrt{r}}f\left(x\right)dx\right)\frac{\lambda (\sqrt{r}-1)}{2(1-(1/r))}+\left(\int_{\sqrt{r}}^{r}f\left(x\right)dx\right)\frac{\lambda (r-\sqrt{r})}{2(1-(1/r))}\\ \; & =\frac{\lambda \left(1-\frac{1}{\sqrt{r}}\right)\left(\sqrt{r}-1\right)}{2{\left(1-\left(1/r\right)\right)}^{2}}+\frac{\lambda \left(\frac{1}{\sqrt{r}}-\frac{1}{r}\right)\left(r-\sqrt{r}\right)}{2{\left(1-\left(1/r\right)\right)}^{2}}\\ & =\frac{\lambda \left(\frac{1}{\sqrt{r}}-\frac{1}{r}\right)\left(r-\sqrt{r}\right)}{{\left(1-\left(1/r\right)\right)}^{2}}\\ & =\frac{\lambda {\left(\sqrt{r}-1\right)}^{2}}{\sqrt{r}{\left(1-\left(1/r\right)\right)}^{2}}. \end{array}$$

□

From the above lemmas, it follows that for λr<1 and large enough *r*,

$$\frac{E[W^{TAGS}\left(s_{T}\right)]}{E[W^{SITA}\left(s^{*}\right)]}\geq \frac{\lambda r}{\frac{\lambda {\left(\sqrt{r}-1\right)}^{2}}{\sqrt{r}{\left(1-\left(1/r\right)\right)}^{2}}}=\frac{{\left(\sqrt{r}+1\right)}^{2}}{\sqrt{r}},$$

where the equality is obtained by simplifying the derived expression. Interestingly, the last expression does not depend on λ. We now show that it is increasing with *r*.

**Lemma** **3.** *The function $\frac{{\left(\sqrt{r}+1\right)}^{2}}{\sqrt{r}}$ is increasing with r.*

**Proof.**  We aim to show that $\frac{{\left(\sqrt{r}+1\right)}^{2}}{\sqrt{r}}$ is increasing with *r*, which is true if and only if

$${\left({\left(\sqrt{r}+1\right)}^{2}\right)}^{\prime }\sqrt{r}-{\left(\sqrt{r}\right)}^{\prime }{\left(\sqrt{r}+1\right)}^{2}>0\Leftrightarrow$$

$$\frac{2(\sqrt{r}+1)}{2\sqrt{r}}\sqrt{r}-\frac{1}{2\sqrt{r}}{\left(\sqrt{r}+1\right)}^{2}>0\Leftrightarrow$$

$$\sqrt{r}+1-\frac{1}{2\sqrt{r}}{\left(\sqrt{r}+1\right)}^{2}>0\Leftrightarrow$$

$$1-\frac{1}{2\sqrt{r}}\left(\sqrt{r}+1\right)>0\Leftrightarrow$$

$$2\sqrt{r}-\left(\sqrt{r}+1\right)>0\Leftrightarrow$$

$$\sqrt{r}-1>0$$

Therefore, the desired result follows. □

From the above results and using that $\frac{{\left(\sqrt{r}+1\right)}^{2}}{\sqrt{r}}$ tends to infinity when r→∞, it follows that when λr<1 and *r* is large, the ratio between the mean waiting time of the TAGS system and that of the SITA system is lower bounded by a function that is unbounded and the following result follows.

**Theorem** **1.** *The ratio of the mean waiting time of the TAGS system and the mean waiting time of the SITA system is unbounded.*

### 4.4. Bounded Pareto with α≠1

We now study the ratio between the mean waiting time of the TAGS system and that of the SITA system for Bounded Pareto distributions with α≠1. We consider the evolution of this ratio when we vary *r* from 10 to 1000 for different values of α.

**Figure 3:**
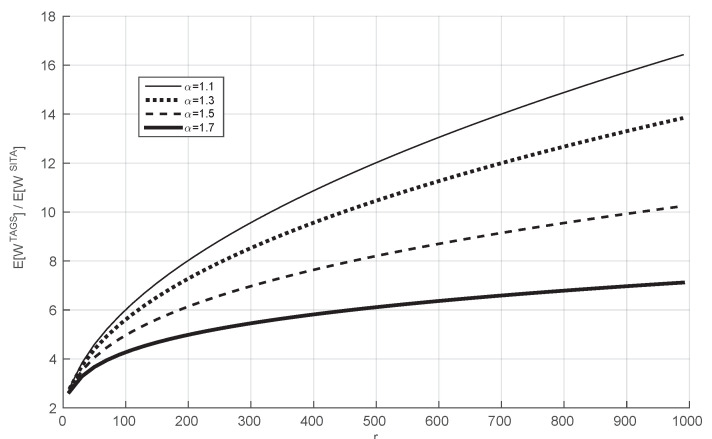
The ratio $E\left[W^{TAGS}\left(s_{T}\right)\right]/E\left[W^{SITA}\left(s_{T}\right)\right]$ as a function of *r* for different values of α. y-axis without units (it is the ratio of two values with the same unit) and for the x-axis the units are ms.

In Figure 3, we consider an arrival rate of 0.001, and we plot the ratio between the mean waiting time of the TAGS system and that of the SITA system when we vary *r* for several values of α larger than one. We observe that the performance ratio under study is increasing with *r* for the considered values of *r*. Therefore, this illustration shows that the result of Theorem 1 generalizes to values of α which are not equal to 1.

## 5. Performance Comparison of TAGS and SITA-E with K≥2 Servers

In this section, we compare the performance of TAGS and SITA-E for an arbitrary number of servers. Specifically, we study the ratio of the mean waiting time of TAGS to that of SITA-E, i.e., $\frac{E[W^{TAGS}\left(s_{T}\right)]}{E[W^{SITA-E}]}$.

Let us denote by $E[W^{SINGLE}\left(\lambda \right)]$ the mean waiting time of a single server with arrival rate λ. We note that the above ratio can be written as

(5) $$\frac{E[W^{TAGS}\left(s_{T}\right)]}{E[W^{SITA-E}]}=\frac{E[W^{TAGS}\left(s_{T}\right)]}{E[W^{SINGLE}\left(\lambda \right)]}\frac{E[W^{SINGLE}\left(\lambda \right)]}{E[W^{SITA-E}]}.$$

We now focus on the term $\frac{E[W^{TAGS}\left(s_{T}\right)]}{E[W^{SINGLE}\left(\lambda \right)]}$. In the following result, we show that, when the arrival rate tends to zero, this ratio tends to one.

**Proposition** **1.** *Let X be a distribution with support in t≥1. When λ→0, then $\frac{E[W^{TAGS}\left(s_{T}\right)]}{E[W^{SINGLE}\left(\lambda \right)]}$ tends to one.*

**Proof.**  A single server system is a special case of a TAGS system with degenerate cut-offs, which implies by the definition of $s_{T}$ that $E\left[W^{SINGLE}\left(\lambda \right)\right]\geq E\left[W^{TAGS}\left(s_{T}\right)\right]$. Let $s_{T,1}$ be the first component of the vector $s_{T}$ and $p(s_{T,1})$ be the probability of jobs larger than $s_{T,1}$. The contribution to the mean waiting time waiting time of TAGS in the first server coming from jobs bigger than $s_{T,1}$ is $p\left(s_{T,1}\right)s_{T,1}\geq p\left(s_{T,1}\right)$, as $s_{T,1}\geq 1$. Therefore, as the waiting time of a single server goes to 0 with λ and, by the definition of $s_{T}$, $s_{T,1}$ is optimal, we conclude that, asymptotically, as λ goes to zero, so does $p(s_{T,1})$, meaning that in the limit all jobs are served and finish in the first server and thus the performance of the system is like that of a single server, i.e., the ratio approaches 1. □

From the above result, we conclude that, when λ→0,

$$\frac{E[W^{TAGS}\left(s_{T}\right)]}{E[W^{SITA-E}]}\to \frac{E[W^{SINGLE}\left(\lambda \right)]}{E[W^{SITA-E}]}.$$

We now analyze the ratio $\frac{E[W^{SINGLE}\left(\lambda \right)]}{E[W^{SITA-E}]}$ when λ→0. For this case, as the mean waiting time of a single server is approximately linear in the arrival rate, it follows that

$$\frac{E[W^{SINGLE}\left(\lambda \right)]}{E[W^{SITA-E}]}\approx \frac{KE[W^{SINGLE}\left(\frac{\lambda }{K}\right)]}{E[W^{SITA-E}]}.$$

The ratio $\frac{E[W^{SINGLE}\left(\frac{\lambda }{K}\right)]}{E[W^{SITA-E}]}$ has been studied in [3]. In that work, the authors conclude that this ratio grows with the variability of the job size distribution and can be large if the variability of the job size distribution is large. For example, from Proposition 3 in [3], we conclude that for Bounded Pareto-distributed job sizes with α=1, the ratio $\frac{E[W^{SINGLE}\left(\frac{\lambda }{K}\right)]}{E[W^{SITA-E}]}$ tends to infinity when the ratio between the shortest and the largest job size tends to zero, and as this result is true for any value of λ, using (5), the next result follows.

**Proposition** **2.** *For Bounded Pareto distributed job sizes with α=1 and K≥2, when λ→0, the ratio between the mean waiting time of TAGS and SITA-E is unbounded from above.*

From this result and using that the mean waiting time of SITA-E is larger than the mean waiting time of the SITA policy with optimal thresholds, the ratio $\frac{E[W^{TAGS}\left(s_{T}\right)]}{E[W^{SITA-E}]}$ is a lower bound of the ratio $\frac{E[W^{TAGS}\left(s_{T}\right)]}{E[W^{SITA}\left(s^{*}\right)]}$. Consequently, we have the following result.

**Corollary** **1.** *For Bounded Pareto-distributed job sizes with α=1 and K≥2, when λ→0, the ratio between the mean waiting time of TAGS and the optimal SITA is unbounded from above.*

In [3], the authors also analyze the performance ratio $\frac{E[W^{SINGLE}\left(\frac{\lambda }{K}\right)]}{E[W^{SITA-E}]}$ for Bounded Pareto distributed job sizes with tail parameter α∈(0,2)\{1} and K→∞, and in Proposition 4, they show that this ratio tends to infinity when the ratio between the shortest and the largest job size tends to zero. Consequently, we obtain the following result

**Proposition** **3.** *For Bounded Pareto distributed job sizes with α∈(0,2)\{1} and K→∞, when λ→0, the ratio between the mean waiting time of TAGS and SITA-E is unbounded from above.*

Furthermore, the corollary below also follows immediately.

**Corollary** **2.** *For Bounded Pareto distributed job sizes with α∈(0,2)\{1} and K→∞, when λ→0, the ratio between the mean waiting time of TAGS and the optimal SITA is unbounded from above.*

## 6. Design of Decentralized Systems

**Figure 4:**
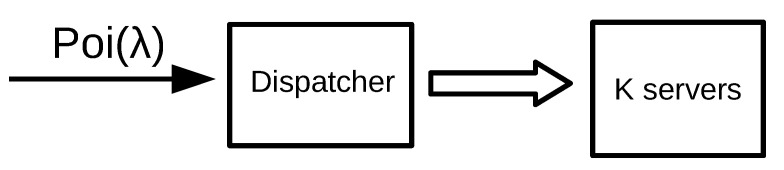
A decentralized system with 2 groups. Each dispatchers receives λ/2 traffic and sends it to K/2 servers.

We consider a decentralized system formed by *n* groups where, in each group, the incoming jobs are routed to K/n servers. In Figure 4, we represent an example of a decentralized system formed by 2 groups. We assume that, in each group, there is a single dispatcher that implements the SITA or TAGS policy and job sizes are Bounded Pareto-distributed.

In a decentralized system with *n* groups, when there are *t* groups that implement the TAGS policy and n−t groups that implement SITA, the performance of the system is given by

$$P\left(t\right)=\frac{t}{n}E\left[W^{TAGS}\left(s_{T}\right)\right]+\left(1-\frac{t}{n}\right)E\left[W^{SITA}\left(s^{*}\right)\right].$$

We assume that there is a quality of service (QoS) condition that states that the average waiting time of the system cannot exceed a given threshold *d*. Therefore, the system designers must decide on a policy that satisfies the condition with minimal cost (i.e., maximize the number of groups that implement the TAGS policy) and satisfies the QoS condition P(t)≤d. This can be expressed as

(6) $$\begin{array}{cc} \max\; & \;t \end{array}$$

(7) $$\begin{array}{cc} \;s.t.\; & P(t)\leq d. \end{array}$$

We denote by $t^{*}$ the solution to the above problem. We assume that P(n)≤d; otherwise, there is no solution to this problem. Given that P(t) is decreasing with *t*, it is clear that if P(0)≤d, then $t^{*}=0$, i.e., all the groups implement the SITA policy. Otherwise, using this monotonicity property, it follows that the solution of this problem is $t^{*}=\left\lfloor P^{-1}\left(d\right)\right\rfloor$. However, finding the analytical value of the inverse of P() is difficult as an analytical expression for the mean waiting time of a system that implements the TAGS policy is not known. As a result, we focus on a suboptimal solution to this problem that uses the result of Theorem 9 in [2] to get insights into $t^{*}$.

We choose the number of groups *n* of the decentralized system so that the load of each group is less than one. As the arrival rate of each group is λ/n, we have that

$$\rho <1\Leftrightarrow n>\frac{E[X]}{\lambda }.$$

We choose the number of groups to be the minimal value of *n* which is a divisor of *K* and is larger than $\frac{E[X]}{\lambda }.$

According to Theorem 9 in [2], when *r* is very large, we have that

$$2E\left[W^{SITA}\left(s^{*}\right)\right]\geq E\left[W\left(s_{T}\right)\right].$$

Thus, for *r* very large, we have that

$$\begin{array}{cc} P(t) & =\frac{t}{n}E\left[W^{TAGS}\left(s_{T}\right)\right]+\left(1-\frac{t}{n}\right)E\left[W^{SITA}\left(s^{*}\right)\right]\\ \; & \leq 2\frac{t}{n}E\left[W^{SITA}\left(s_{T}\right)\right]+\left(1-\frac{t}{n}\right)E\left[W^{SITA}\left(s^{*}\right)\right]\\ & =\left(1+\frac{t}{n}\right)E\left[W^{SITA}\left(s^{*}\right)\right]. \end{array}$$

Let $\tilde{P}\left(t\right)=\left(1+\frac{t}{n}\right)E\left[W^{SITA}\left(s^{*}\right)\right].$ Therefore, an alternative optimization problem to (6) and (7) consists of considering the constraint $\tilde{P}\left(t\right)\leq d$ instead of P(t)≤d, i.e.,

(8) $$\begin{array}{cc} \max\; & \;t \end{array}$$

(9) $$\begin{array}{cc} \;s.t.\; & \tilde{P}\left(t\right)\leq d. \end{array}$$

The solution of this problem is clearly the minimum between *n* and $\left\lfloor {\bar{t}}^{\;*}\right\rfloor ,$ where ${\bar{t}}^{\;*}$ is such that $\tilde{P}\left(t^{\;*}\right)=d$, i.e.,

$$\left(1+\frac{{\bar{t}}^{\;*}}{n}\right)E\left[W^{SITA}\left(s^{*}\right)\right]=d\Leftrightarrow {\bar{t}}^{\;*}=n\left(\frac{d}{E[W^{SITA}\left(s^{*}\right)]}-1\right).$$

Finally, we can obtain the expression of $E[{\bar{W}}^{SITA}\left(s^{*}\right)]$ for Bounded Pareto-distributed job sizes with large *r* and ρ<1 using the formula of Theorem 6.3 in [16].

### Example

We consider an example with a decentralized system in which $r=10^{4}$ and α=1.25. For this case, we obtain that E[X]=27.027. Thus, in a system formed by 1000 servers and arrival rate equal to 5,

$$\frac{\lambda E[X]}{n}=\frac{5\cdot 27.027}{n}<1\Leftrightarrow n>135.135.$$

As 200 is the minimum divisor of 1000 that verifies the condition of the last expression, we assume that the decentralized system is formed by a number of groups equal to 200. This number of groups meets the condition that establishes that the load of each server is less than one:

$$\frac{\lambda E[X]}{n}=\frac{5\cdot 27.027}{200}=0.675<1.$$

Using Theorem 6.3 in [16], we get that the normalized mean waiting time of a SITA system formed by 5 servers with $r=10^{4}$, α=1.25, and ρ=0.675 is given by 0.216. Therefore, if the limit *d* of the model is set to 0.4, we get for our example that

$${\bar{t}}^{\;*}=200\left(\frac{0.4}{0.216}-1\right)=170.28.$$

This result means that if 170 groups of 5 servers implement the TAGS policy and 30 groups implement the SITA policy, we can ensure that the quality of service condition (7) is satisfied.

## 7. Conclusions

We have studied the performance of parallel server systems with two size-based task assignment policies: SITA and TAGS. Both policies use cut-offs to determine how jobs are executed in the servers. The main difference between them is that SITA uses the size of incoming tasks to assign jobs to servers, while TAGS does not.

The goal of this paper is to analyze the ratio between the mean waiting time of TAGS and the mean waiting time of SITA. This ratio can be seen as the penalty for not knowing the size of incoming tasks. In a previous work [2], we have shown that this ratio is upper bounded by 2 when the system load is fixed and less than one and the ratio between the largest and the shortest job tends to infinity. In this article, we extend this result in several directions.

We first consider a non-asymptotic regime, and we compare the performance of SITA and TAGS for this regime. For Bounded Pareto-distributed job sizes with tail parameter 1 and two servers, we provide a lower bound on the mean waiting time of TAGS and an upper bound of the mean waiting time of SITA, in both cases assuming that the arrival rate times the largest job size is smaller than one. From these results, we provide a lower bound of the ratio of the mean waiting time of TAGS over the mean waiting time of SITA of a system with two servers and in the considered regime. We show that this lower bound has nice properties, for example, it does not depend on the arrival rate and it is increasing with the size of the largest job. We conclude that the mean waiting time of TAGS divided by the mean waiting time of SITA is unbounded from above in a system with two servers and when the arrival rate time of the largest job size is smaller than one. We also explore numerically this performance ratio when the tail parameter is not 1, and we observe that our theoretical findings are not limited to Bounded Pareto-distributed job sizes with tail parameter 1.

Then, we analyze a system with an arbitrary number of servers, and we study the ratio of the mean waiting time of TAGS under the mean waiting time of SITA-E. We consider the light traffic regime in which the arrival rate tends to zero. For this regime, we first show that the mean waiting time of TAGS and the mean waiting time of a single server coincide. From this result, it follows that the performance ratio under consideration is proportional to the performance ratio that the authors of [3] explored. This similitude lets us conclude that the ratio of the mean waiting time of TAGS to that of SITA-E is unbounded from above for Bounded Pareto distributed job sizes with α=1 and K≥2, and for Bounded Pareto distributed job sizes with α∈(0,2)\{1} and K→∞.

We would like to remark that the above results show that the parameters of the system can be set in a way that the mean waiting time of TAGS can be much worse than the mean waiting time of SITA, and therefore the penalty for not knowing the job size is unbounded from above.

In this work, we also consider decentralized systems in which the system is divided into groups, where each group handles the same amount of traffic and sends the traffic to a subset of dedicated servers. Each group can operate under SITA or TAGS. The goal is to find the maximum number of groups that can operate under TAGS policy and still satisfy a given quality of service condition. We provide a suboptimal solution to this problem using the asymptotic result in [2].

The analysis presented in this article assumes several properties of Bounded Pareto distributions. A possible extension of this work would be to consider an arbitrary distributions and to compare the performance of other popular routing policies from the literature such as Join the Shortest Queue and Power of Two. Finally, we are interested in studying the application of learning techniques to parallel server systems using the approach in [23] and the interaction between the techniques presented in this work with other control methodologies such as fuzzy control or Active Disturbance Rejection Control as in [24].

## Data Availability

Not applicable.
